# SHoRT-CA: Smart home data repository of time series with contextual anomalies

**DOI:** 10.1016/j.dib.2026.113370

**Published:** 2026-10-22

**Authors:** Aafan Ahmad Toor, Jia-Chun Lin, Ernst Gunnar Gran

**Affiliations:** a Department of Information Security and Communication Technology, Norwegian University of Science and Technology, Teknologivegen 22, Gjøvik, 2815, Innlandet, Norway; b Simula Research Laboratory, Kristian Augusts gate 23, Oslo, 0164, Norway

**Keywords:** Internet of things, Indoor air quality, Environmental sensing, Anomaly detection, Sensor data

## Abstract

Indoor air quality can be affected by airborne harmful particles and materials, which can result in short-term and long-term health issues. Healthy indoor air quality plays a critical role in maintaining a healthy environment in an Internet of Things (IoT) based smart home. Detecting any anomalies in indoor air quality is helpful to minimize health risks and improve the quality of life. Contextual anomalies are a type of deviation in data that are bound to a temporal context, i.e., without the context, anomalies cannot be separated from the normal data. To the best of our knowledge, publicly available multivariate indoor air quality datasets with explicitly documented contextual anomalies remain limited. This study presents an openly available data repository named Smart Home Data Repository of Time series with Contextual Anomalies (SHoRT-CA), comprising multivariate time series data measuring indoor air quality in a smart home. The repository comprises multiple datasets, including data values of air quality, human activity, and environmental interaction, which contain data recorded at different time intervals and periods. The contextual anomalies are induced into the datasets by performing various experiments to affect the sensors. The repository consists of data files, anomaly descriptions, and figures of sensor values and highlighted anomalies. The repository supports research on contextual anomaly detection in IoT-based smart home environments by providing labeled anomaly events alongside detailed sensor measurements for method development and evaluation.

Specifications Table

**Table:** 

Subject	Computer Sciences
Specific subject area	*IoT-based indoor air quality monitoring and contextual anomaly detection*
Type of data	*CSV files (Raw, Time series, Tabular)*
Data collection	*Multivariate sensor data were collected using Airthings View Plus and Space Hub devices from an office, kitchen, and bedroom. The sensors recorded temperature, humidity, CO₂, PM1, PM2.5, PM10, VOC, pressure, sound, and light, with additional virtual sensors for virus risk, occupants, and presence. Data were exported as CSV files at 2.5–60 min intervals.*
Data source location	*Gjøvik, Innlandet, Norway*
Data accessibility	Repository name: Zenodo Data identification number: 10.5281/zenodo.18504688 Direct URL: https://doi.org/10.5281/zenodo.18504688 Instructions: Download the ZIP file in the repository [1] and follow the included folder structure and anomaly-details file to access and interpret the data.
Related research article	Toor, A.A., Lin, J.-C., Gran, E.G., Lee, M.-C.: Uocad: An unsupervised online contextual anomaly detection approach for multivariate time series from smart homes. In: IoTBDS 2024: Proceedings of the 9th International Conference on Internet of Things, Big Data and Security, April 28-30, 2024, Angers, France (2024) [2].

## 1 Value of the Data

• To the best of our knowledge, SHoRT-CA is among the first publicly available datasets to provide real-world multivariate indoor air quality measurements with explicitly labeled contextual anomalies from smart home environments.
• The dataset provides multivariate indoor environmental measurements, including temperature, humidity, CO₂, particulate matter, VOC, pressure, sound, and light, together with occupancy and presence information.
• The dataset includes documented contextual anomaly events generated in realistic indoor environments, providing a resource for developing and evaluating contextual anomaly detection methods.
• The combination of environmental and contextual variables enables researchers to investigate relationships between indoor conditions, occupancy, and human activities in IoT-based smart environments.
• The dataset can support research on multivariate time series analysis, indoor air quality monitoring, sensor correlation, and exploratory anomaly detection in smart homes and related IoT environments.

## 2 Background

Smart home environments increasingly rely on Internet of Things (IoT)-based environmental sensors to monitor indoor air quality (IAQ), generating large volumes of multivariate time series data. Indoor environmental conditions are strongly influenced by occupancy, human activities, ventilation, and other contextual factors, resulting in deviations from normal patterns, referred to as anomalies. Anomalies in time series data are commonly categorized as point anomalies (deviation of a single observation), collective anomalies (deviation of multiple consecutive observations), and contextual anomalies (anomaly only with respect to its surrounding context). While point and collective anomalies can often be identified from the statistical properties of the observations, contextual anomalies are abnormal only with respect to a particular temporal, environmental, behavioral, or operational context. For instance, while spikes in CO_2_ and particulate matter are expected routine events during cooking, similar spikes during non-cooking hours can cause contextual anomalies. Effectively detecting these events requires models capable of learning long-term dependencies across multiple co-dependent environmental variables, making multivariate contextual anomaly detection critical for accurate IAQ health and safety monitoring.

It is easy to identify an outlier as an anomaly by comparing it with a pre-defined threshold and labeling it without knowing its context, but labeling contextual anomalies requires domain expertise and awareness of the events that caused the anomaly. Existing studies employ several strategies to obtain/generate anomalous data [3], including generating naturally occurring or observational anomalies [[4], [5], [6], [7], [8]], generating synthetically injected anomalies, and generating anomalies caused by controlled or semi-controlled experiments [[9], [10], [11], [12], [13], [14]]. Naturally occurring anomalies can provide highly realistic observations, but obtaining a sufficiently large quantity of well-labeled events can be difficult. Synthetic injection provides greater control over anomaly characteristics and labels, but the injected patterns may not fully represent real-world events [15]. Controlled or semi-controlled experiments, i.e., used in this study, provide an alternative by deliberately inducing real-world events while retaining control over their timing and context. There are many point and contextual anomaly datasets, but there is a scarcity of smart home time series datasets with contextual anomalies.

According to the literature review for this study, no openly available multivariate time series indoor air quality dataset from smart homes provides labeled contextual anomalies from real-world or semi-controlled settings. A comparison of existing datasets with SHoRT-CA is provided in Table 1. Table 1 summarizes the characteristics of the evaluated datasets across six key dimensions: dataset name, operational domain, multivariate nature, and the anomaly generation strategy. It also specifies whether the datasets include contextual anomalies linked to environmental or operational settings and verifies whether they are openly available to the research community. The “Contextual anomalies?” column in Table 1 indicates whether the source explicitly reports or labels anomalies according to their temporal, environmental, behavioral, or operational context. “Not reported” indicates that contextual anomalies are not explicitly described or labeled in the cited dataset publication. “Partial” indicates that contextual or behavioral information is available or can be inferred, but contextual anomalies are not explicitly labeled. We discuss some of the existing datasets that either have anomalies that can be classified as contextual anomalies, or they generally fall in the domain of IoT, smart home, or indoor air quality. A study conducted for time series anomaly detection proposes an indoor air quality dataset [4] that measures CO2 levels from multiple schools in New Zealand. The dataset reports anomalies in the form of fluctuations in the CO2 levels during the time the classrooms are occupied by the students. However, this dataset is neither multivariate nor openly available. Two air quality datasets comprise readings from indoor air quality in an airport [5] and urban air quality [6], with pollution events as anomalies. Both these datasets are openly available, but the type of anomalies they offer is not contextual. Another dataset [9] comprises environmental, energy consumption, and network data from a smart home. Two actors performed various activities in the home to generate cyber-physical anomalies. The dataset is not openly available; however, it can be made available upon request.

**Table 1:** Comparison of SHoRT-CA with existing datasets

Dataset	Domain	Multivariate?	Anomaly generation strategy	Contextual anomalies?	Openly available?
Dunedin CO₂ [4]	Indoor air quality / schools	No	Natural / Observational	Yes	No
Indoor airport air quality [5]	Indoor air quality / airport	Yes	Natural / Observational	Not reported	Yes
AirWare Haikou [6]	Urban air quality	Yes	Natural / Observational	Not reported	Yes
CUBRE [9]	Smart home / CPS	Yes	Controlled / Semi controlled	Partial	On request
SWaT [10]	Water treatment / CPS	Yes	Controlled	No	On request
WaDi [11]	Water distribution / CPS	Yes	Controlled	No	On request
GECCO [12]	Drinking water quality	Yes	Controlled	Not reported	Yes
CPS subsystem [13]	Liquid storage / distribution	Yes	Controlled	No	Yes
IoT-DH [14]	IoT network security	Yes	Controlled	No	Yes
SMD [7]	IT / Server infrastructure	Yes	Natural / observational	No	Yes
Smart building [16]	Smart building	Yes	Not reported	Not reported	Yes
IoT IAQ [17]	Indoor air quality	Yes	Not reported	Not reported	On request
Low-cost smart home IAQ platform [8]	Smart home / IAQ	Yes	Natural / observational	Not reported	On request
CU-BEMS [18]	Smart building	Yes	Not reported	Not reported	Yes
SHoRT-CA	Smart home / Office / IAQ	Yes	Semi controlled	Yes	Yes

Many datasets from the industrial IoT domain have been used for anomaly detection and other tasks. Two of these datasets, named SWaT [10] and WaDi [11], are considered benchmarks in the anomaly detection domain. SWaT and WaDi provide support for secure Cyber-Physical Systems (CPS) by recording the data from the water treatment and distribution testbed, respectively, and contain cyber attacks as anomalies. GECCO [12] is another dataset from the industrial IoT domain that measures the drinking water quality using various sensors and considers water contamination events as anomalies. Another industrial IoT dataset [13] records raw sensor measurements from a liquid storage and distribution cyber-physical subsystem. The anomalies represent cyber attacks, human errors, and environmental factors causing fluctuations in more than one sensor. IoT-DH [14] and SMD [7] are two datasets from IT networks and infrastructure monitoring areas that contain cyber attacks and server failures as anomalies. Monitoring of smart homes and buildings for energy consumption and air quality is another area where many openly available datasets are available. Some of these datasets [16,17,8,18] do not contain anomalies; however, they offer sensor data and insights into the circumstances where the data was collected. Hence, these datasets can be custom-labeled for unusual patterns, fluctuations in air quality, and human activities, etc., and used for an anomaly detection task. Recent work has also demonstrated the use of multivariate time series data and LSTM-based models for building energy-consumption forecasting, highlighting the broader role of temporal environmental data in data-driven building management [19]. Anomaly detection is also an important research area in IoT security, where deep-learning approaches have been investigated for detecting malicious or abnormal network activity [20].

In this study, we present SHoRT-CA, i.e., **S**mart **Ho**me data **R**epository of **T**ime series with **C**ontextual **A**nomalies. In comparison with the other publicly available datasets, SHoRT-CA is openly available and contains contextual anomalies induced by performing semi-controlled experiments involving indoor air quality sensors. The anomaly contexts are clearly defined and generated from clearly defined experiments to mimic real-world events in a smart home. A thorough description of how the datasets were collected and labeled is provided in the following sections. The datasets can benefit researchers working on anomaly detection, IoT-based environmental monitoring, and smart building analytics, as well as developers designing data-driven monitoring systems for indoor environments. The repository aims to support research on contextual anomaly detection in smart home environments by providing multivariate sensor data together with well-documented contextual events. Some parts of these datasets have recently been used to effectively detect contextual anomalies in an unsupervised and online manner by proposing UoCAD [2] and UoCAD2 [21] methods. While the dataset captures realistic indoor sensing scenarios, it is collected from a limited number of locations and is intended primarily for methodological research on anomaly detection.

## 3 Data Description

This section describes the SHoRT-CA data repository, including the collection environments, sensor measurements, contextual anomalies, and organization of the available data records.

### 3.1 Overview of the data

Fig. 1 gives an overview of the data by visualizing the sensor values for the first data collection period, i.e., P1. P1 comprises data from ten physical and three virtual sensors. It is to be noted that the data collection period is the same for all sensors, i.e., from 2023-01-12 to 2023-02-16; the instance counts vary for each sensor because of the variable intervals. Similar figures for the rest of the data collection periods are included in the data repository. The data visualizations might show some point and collective anomalies, e.g., the latter part of the presence and occupancy sensors in Fig. 1. These anomalous behaviors were not part of the planned experiments but occurred naturally due to unknown causes. We leave this for the users to consider these occurrences as unknown anomalies.

**Fig. 1:**
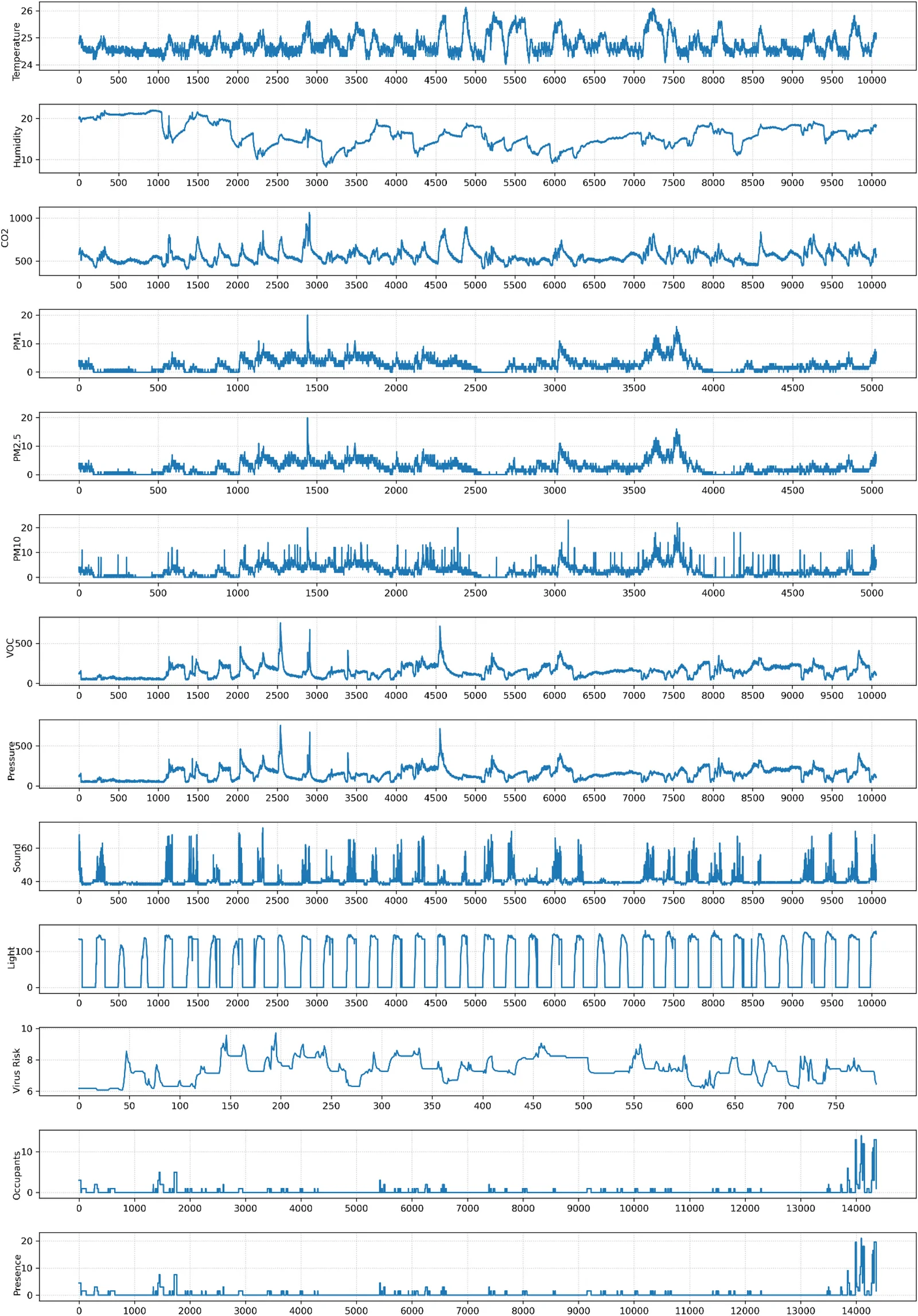
Visualization of the thirteen sensors for the data collection period of P1. (The x-axis represents instance count and the y-axis represents respective sensor values)

### 3.2 Sensor measurements

The SHoRT-CA repository is designed to support research on contextual anomaly detection in multivariate time series data from smart home environments, particularly for indoor air quality monitoring and IoT-based sensing applications. The dataset includes measurements from multiple environmental sensors along with clearly documented contextual anomaly events. The summary of the physical and virtual sensors, i.e., sensor ID, period ID, total instances, and interval, is provided in Table 2, Table 3. These tables also display the statistics, i.e., min, max, average (denoted by μ), and standard deviation (denoted by σ). These statistics show the variations among different sensors as well as different data periods within the same sensor. Sensors S1 to S11 recorded data for all seven periods, but with varying numbers of instances and intervals of 2.5, 5, and 60 minutes. Sensors S12 and S13 only recorded data for one data collection period, i.e., P1, having 14,364 instances and an interval of 5 minutes. The different sampling intervals result from the device configuration and sensor design. View Plus operates at 5-minute intervals when battery-powered and can operate at 2.5- or 5-minute intervals when connected to USB-C power, while the virus risk virtual sensor is by design calculated at 60-minute intervals. Consequently, analyses requiring synchronized multivariate observations may require resampling or aggregation to a common temporal resolution.

**Table 2:** Summary and statistics of the different collection periods of the physical sensors

Sensor ID	Period ID	Instance Count	Interval (minutes)	Statistics			
				Min	Max	μ	σ
S1	P1	10,059	5.0	24.0	26.1	24.7	0.3
	P2	18,225	5.0	19.4	27.0	23.3	0.9
	P3	18,114	2.5	12.6	32.9	22.1	2.6
	P4	17,787	2.5	9.3	19.8	14.8	1.9
	P5	13,273	2.5	22.5	24.8	23.3	0.4
	P6	6,395	2.5	-6.2	24.1	13.5	3.3
	P7	120,210	2.5	9.0	39.0	24.7	2.7
S2	P1	10,059	5.0	8.2	22.0	15.9	2.8
	P2	18,225	5.0	25.3	80.9	45.4	6.9
	P3	18,114	2.5	27.2	95.9	51.2	9.3
	P4	17,787	2.5	37.6	90.5	69.4	8.6
	P5	13,273	2.5	7.2	22.9	16.7	3.2
	P6	6,395	2.5	25.2	94.5	68.2	7.0
	P7	120,210	2.5	18.1	96.4	44.0	8.8
S3	P1	10,059	5.0	400	1,064.0	554.2	75.8
	P2	18,225	5.0	417	2,350.0	695.7	321.4
	P3	18,114	2.5	427	4,001.0	1,441.8	463.1
	P4	17,787	2.5	400	3,424.0	1,456.3	489.2
	P5	13,273	2.5	400	1,098.0	525.8	92.8
	P6	6,395	2.5	400	3,873.0	1,593.1	467.5
	P7	120,210	2.5	370	2,565.0	926.5	409.5
S4	P1	5,028	5.0	0	20.0	2.6	2.3
	P2	7,553	5.0	0	611.0	26.0	56.7
	P3	18,114	2.5	0	685.0	77.5	97.5
	P4	17,787	2.5	0	461.0	66.4	77.7
	P5	13,274	2.5	0	17.0	2.5	2.8
	P6	6,395	2.5	0	487.0	78.8	97.8
	P7	120,175	2.5	0	706.0	38.1	81.5
S5	P1	5,028	5.0	0	20.0	2.6	2.3
	P2	7,553	5.0	0	1,000.0	27.2	61.3
	P3	18,114	2.5	0	1,000.0	81.9	110.3
	P4	17,787	2.5	0	599.0	70.1	84.2
	P5	13,274	2.5	0	17.0	2.5	2.8
	P6	6,395	2.5	0	652.0	87.8	115.8
	P7	120,171	2.5	0	1,000.0	42.9	103.2
S6	P1	5,028	5.0	0	23.0	2.7	2.6
	P2	7,553	5.0	0	1,000.0	31.6	74.2
	P3	18,114	2.5	0	1,000.0	93.9	132.1
	P4	17,787	2.5	0	1,000.0	84.1	111.1
	P5	13,273	2.5	0	28.0	2.7	3.0
	P6	6,395	2.5	0	1,000.0	120.2	181.0
	P7	120,172	2.5	0	1,000.0	52.9	131.4
S7	P1	10,059	5.0	46.0	757.0	152.0	75.9
	P2	18,223	5.0	46.0	1,819.0	242.4	181.6
	P3	9,056	5.0	46.0	1,783.0	228.7	220.7
	P4	18,893	5.0	46.0	1,448.0	203.8	181.7
	P5	6,637	5.0	46.0	597.0	182.9	80.1
	P6	3,197	5.0	46.0	2,079.0	296.6	348.5
	P7	60,086	5.0	46.0	4,851	194.5	198.7
S8	P1	10,059	5.0	950	1,015.0	989.6	18.4
	P2	18,223	5.0	958	1,007.0	988.5	10.0
	P3	9,056	5.0	965	1,006.0	987.7	10.1
	P4	18,893	5.0	963	1,008.0	992.5	9.4
	P5	6,637	5.0	945	1,016.0	985.9	18.6
	P6	3,197	5.0	947	997.0	973.9	11.1
	P7	60,086	5.0	946	1,015.0	990.8	9.5
S9	P1	10,059	5.0	38.0	72.0	40.7	4.1
	P2	9,870	5.0	37.0	88.0	47.0	8.8
	P3	18,114	2.5	37.0	94.0	51.3	12.0
	P4	17,787	2.5	37.0	80.0	43.3	6.6
	P5	13,274	2.5	37.0	78.0	40.7	4.8
	P6	6,395	2.5	38.0	79.0	44.8	7.4
	P7	120,188	2.5	37.0	87.0	53.3	11.3
S10	P1	10,059	5.0	0	158.0	53.5	65.4
	P2	18,224	5.0	0	221.0	63.5	63.2
	P3	18,114	2.5	0	161.0	24.2	29.2
	P4	17,787	2.5	0	71.0	0.6	3.6
	P5	13,273	2.5	0	156.0	39.7	59.0
	P6	6,395	2.5	0	122.0	2.3	9.8
	P7	120,210	2.5	0	143.0	45.3	40.3

**Table 3:** Summary and statistics of the different collection periods of the virtual sensors

Sensor ID	Period ID	Instance Count	Interval (minutes)	Statistics			
				Min	Max	μ	σ
S11	P1	791	60	6.1	9.7	7.4	0.7
	P2	1,521	60	1.0	10	4.1	2.5
	P3	759	60	4.4	10	8.7	1.5
	P4	741	60	4.0	10	9.1	1.4
	P5	554	60	5.6	10	7.5	1.0
	P6	270	60	6.0	10	9.6	0.8
	P7	5,012	60	1.0	10	6.2	2.5
S12	P1	14,364	5	0	14.0	0.3	1.3
S13	P7	14,364	5	0	21.0	0.4	1.9

### 3.3 Contextual anomalies

This study focuses on datasets with contextual anomalies; semi-controlled experiments were conducted to induce these anomalies into the data. Potentially, many activities can cause contextual anomalies, e.g., fire causes a sudden spike in particulate matter and VOC, and moderately affects temperature, humidity, and CO_2_. Similarly, the use of cleaning products, paints, and other chemicals with closed ventilation can drastically deteriorate the indoor air quality. However, these activities cannot be performed in a safe and controlled way with humans present in the premises. Hence, the experiments in this study are carefully designed by performing certain activities near the sensors that cause contextual anomalies without compromising the health and safety of the occupants.

Before explaining the contextual anomalies, it is important to clarify the baseline behavior of indoor air quality sensors. Under normal operating conditions, the indoor air quality sensors exhibit stable temporal patterns reflecting routine household or office activities. Temperature and humidity typically vary gradually throughout the day due to heating, ventilation, and outdoor weather conditions. CO_2_ levels generally increase during periods of human presence and decrease when rooms are unoccupied or ventilated. Particulate matter (PM1, PM2.5, and PM10) and VOC values remain relatively low during normal activities but may show short-lived increases during cooking or temporary environmental disturbances. Sound and light sensors reflect occupancy-related activity patterns, typically showing higher values during daytime and lower levels at night. These baseline behaviors can be observed in the visualizations presented in Fig. 1 and are further reflected in the statistical summaries reported in Table 2, Table 3. Contextual anomalies in SHoRT-CA correspond to deviations from these typical patterns occurring under unusual temporal or environmental contexts.

Five activities, i.e., heating malfunction, unintended cooking, ventilation malfunction, attempted burglary, and large gathering, were chosen because of their possible impact on the sensors. Some of these activities were performed multiple times at different locations and during different seasons to generate contextual anomalies of varying nature. Table 4 provides the anomaly details, such as anomaly ID (A1 to A11), period ID, anomaly type (denoted by the initials of the anomaly type), anomaly duration, and a list of sensors that were affected by the anomaly. One example in Fig. 2 visualizes the A2 anomaly highlighted in red, where the sensor readings from the day when the anomaly occurred are displayed. The rest of the visualizations of other anomalies can be found in the figures→anomalies folder in the main data repository [1]. Further details of each type of contextual anomaly are provided in the following sections.

**Table 4:** Details of the anomalies

Anomaly ID	Type	Period ID	Duration	Sensors
A1	HM	P3	2023-10-31 10:00 - 2023-10-31 11:30	S1, S2, S3, S4, S5, S6, S7
A2	UC	P3	2023-11-03 08:30 - 2023-11-03 09:30	S1, S2, S3, S4, S5, S6, S7
A3	PE	P3	2023-11-04 18:00 - 2023-11-04 23:00	S1, S2, S3, S4, S5, S6, S7, S8, S9, S10
A4	AB	P3	2023-11-09 23:45 - 2023-11-10 00:00	S9, S10
A5	HM	P4	2023-11-24 18:00 - 2023-11-24 20:30	S1, S2, S3, S4, S5, S6, S7, S8
A6	VM	P4	2023-11-26 13:00 - 2023-11-26 15:00	S2, S3, S4, S5, S6, S11
A7	HM	P6	2024-01-16 11:00 - 2024-01-16 13:00	S1, S2, S3, S10, S11
A8	VM	P6	2024-01-16 13:00 - 2024-01-16 15:00	S4, S5, S6, S7, S8
A9	HM	P6	2024-01-22 13:30 - 2024-01-22 15:30	S1, S2, S3, S4, S5, S6, S11
A10	HM	P7	2024-02-14 10:00 - 2024-02-14 12:00	S1, S2, S3, S10, S11
A11	UC	P7	2024-02-16 10:00 - 2024-02-16 11:00	S1, S2, S3, S4, S5, S6, S7, S8, S9

**Fig. 2:**
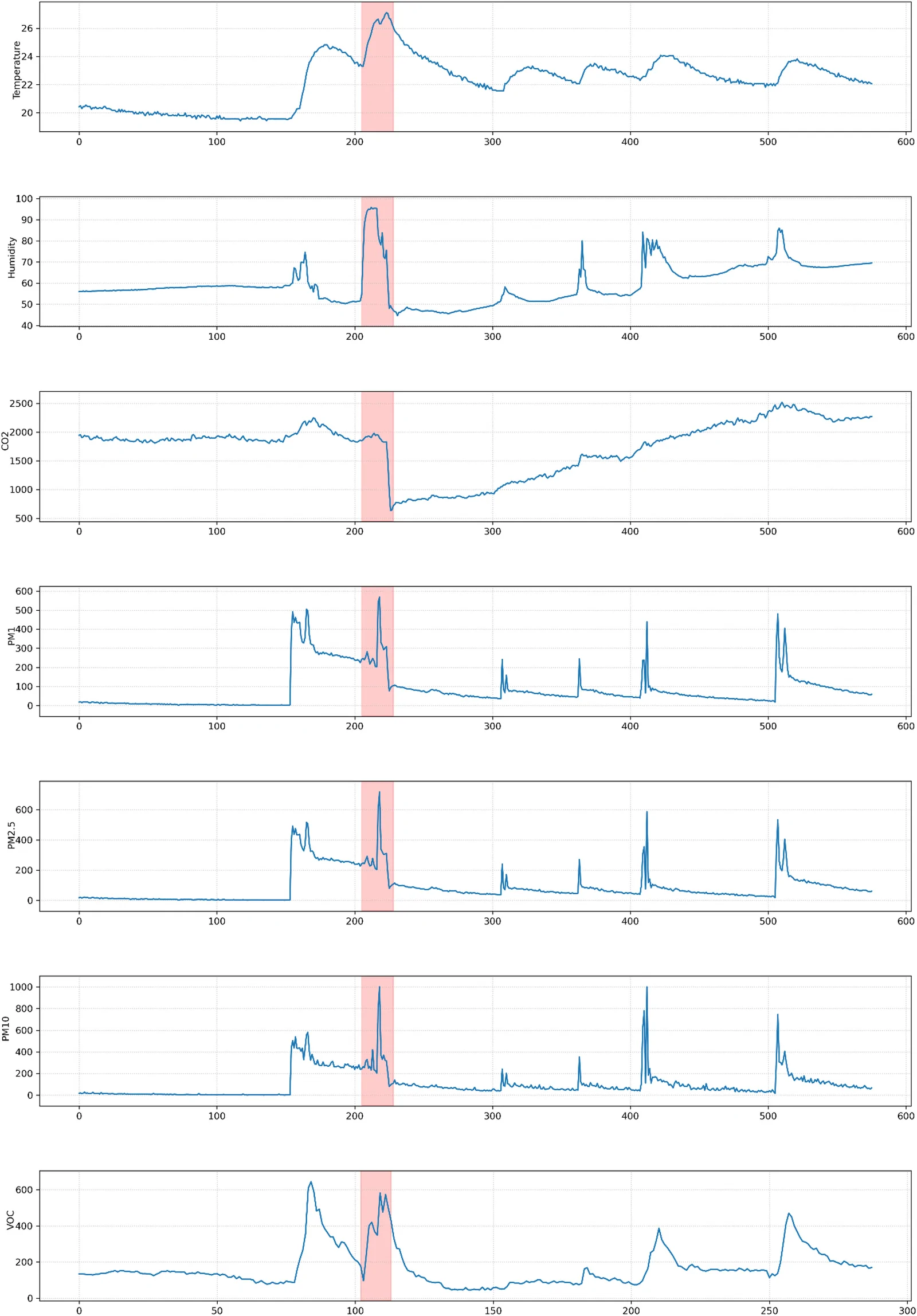
Visualization of the seven sensors involved in A2.

#### 3.3.1 Heating malfunction (HM)

The heating malfunction experiment was designed to simulate a scenario where indoor heating stopped working. The autumn/winter season was chosen for this activity so that the impact of outside weather would be maximum on the indoor air quality. During the experiment, indoor heating was turned off, and the doors and windows were opened. Heating malfunction experiments were performed five times during different data collection periods; the resultant contextual anomalies are referred to as A1, A5, A7, A9, and A10 in Table 4. Variations in the outside temperature have differently impacted the deviations in the sensor values for each anomaly, which can be seen in the Sensors column of Table 4.

#### 3.3.2 Unintended cooking (UC)

This experiment was performed in the kitchen, and it simulated the unintended cooking activity. The context is as if someone mistakenly left the stove on with food on it. This activity was performed at a time when no cooking activity is usually done. During the experiment, the stove was turned on, and a pot containing vegetables was left on the stove. The ventilation (exhaust fan) was turned off during the experiment so that the smoke generated from the cooking had maximum impact on the sensors. Unintended cooking was performed twice, and two contextual anomalies, i.e., A2 and A11, were generated from these experiments.

#### 3.3.3 Party/Event (PE)

Party/Event, as the name suggests, is a contextual anomaly that simulates the presence of a large gathering in the room where the sensors are placed. The experiment was conducted in the living room of the house, with the sensors placed in the adjacent open kitchen. For the experiment, 15 people (seven adults and eight children) were invited to attend a party, and they stayed in the room for 4-5 hours. The contextual anomaly generated from this experiment is referred to as A3 in Table 4.

#### 3.3.4 Attempted burglary (AB)

This experiment was performed to simulate a burglary in the house. For the experiment, the lights were turned on, and noise was made near the sensor for a short duration. The time of the experiment was chosen when the tenants are usually asleep, and no light or noise is made. The resultant anomaly, referred to as A4 in Table 4, affected the light and sound sensors only. About the realism of this experiment, it can be argued that a burglar may not always turn the lights on during a burglary; however, depending on the circumstances, we consider this scenario as one of the many possibilities that can happen during a burglary.

#### 3.3.5 Ventilation malfunction (VM)

This contextual anomaly represents an event in which the ventilation is turned off or stops working. The experiment was conducted by closing all doors, windows, and exhausts in the room and turning the heating to maximum. The idea was to trigger a sudden increase in particulate matter, pressure, and VOC. This experiment was repeated two times, and contextual anomalies A6 and A8 were generated.

### 3.4 Data organization and file structure

The datasets are made available in the form of a zip file in the Zenodo repository [1]. Fig. 3 shows the folder and file organization of the main data repository. Data from each sensor is separately stored in a folder, and folder names are the same as the respective sensor names. CSV files within the sensor folder contain period ID and location ID in their names. All the figures used in this paper, as well as the figures of all anomalies, are provided in the figures folder. A text file named *anomaly_details* contains all the information needed to label the contextual anomalies in different data collection periods.

**Fig. 3:**
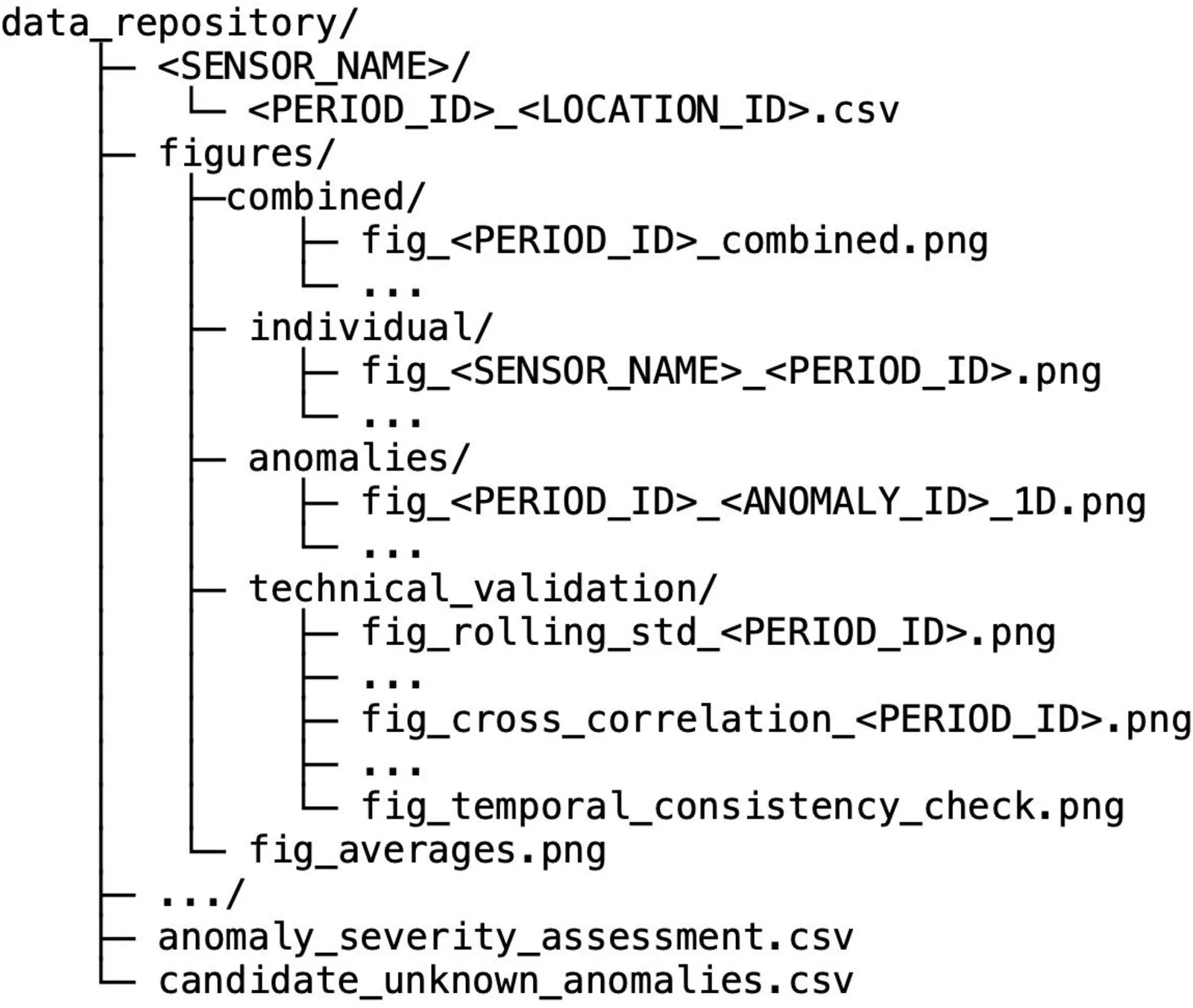
Folder and file organization of the data repository.

Each CSV file consists of two columns, i.e., 'timestamp' and 'value'. The 'timestamp' column is of the datetime type and contains the chronological timestamp during a certain data collection period. The 'value' column is of double type and contains the sensor values associated with a specific timestamp. Table 5 shows the column details, including the format of the timestamps.

**Table 5:** Description of the columns in each data file.

Column Name	Description	Data Type
timestamp	Timestamp when the sensor collected the data. **Format:** YYYY-MM-DDThh:mm:ss	date time
value	Raw sensor value	double

### 3.5 Technical validation

SHoRT-CA datasets are technically validated in this section by checking the temporal consistency, local variability and noise assessment, cross-variable consistency, anomaly severity assessment, and statistical screening of candidate naturally occurring deviations. Together, these analyses evaluate stability over time, short-term signal behavior, and logical relationships among variables. During the data collection process, we observed that environmental sensors exhibit different levels of variability depending on occupancy patterns, ventilation conditions, and seasonal factors, which should be considered when developing and evaluating anomaly detection models using this dataset.

**Temporal Consistency Check:** Temporal consistency was assessed by computing the inter-sample interval between consecutive timestamps for each dataset, as shown in Fig. 4. Periods 1–2 show a dominant sampling interval of approximately 300 seconds, while periods 3–7 are centered around 150 seconds, consistent with the configured acquisition rates. The interval distributions are strongly unimodal with low variance, particularly for period 4, where intervals remain tightly clustered between 146–155 seconds, indicating highly stable sampling with minimal jitter. No duplicated timestamps or negative intervals were detected. The time-series view confirms regular periodic sampling across the majority of the recorded intervals. A small number of isolated extended gaps are visible in periods 2, 3, 6, and 7, likely reflecting temporary communication or logging interruptions. The isolated extended gaps should be considered when applying methods that assume continuous and regularly sampled observations. Depending on the analysis, users may exclude affected intervals, explicitly represent missing observations, or apply an appropriate imputation or resampling strategy. These events are infrequent relative to the total recording duration and do not indicate systematic data loss. Overall, the results demonstrate consistent periodic sampling and preservation of raw temporal structure across all recordings.

**Fig. 4:**
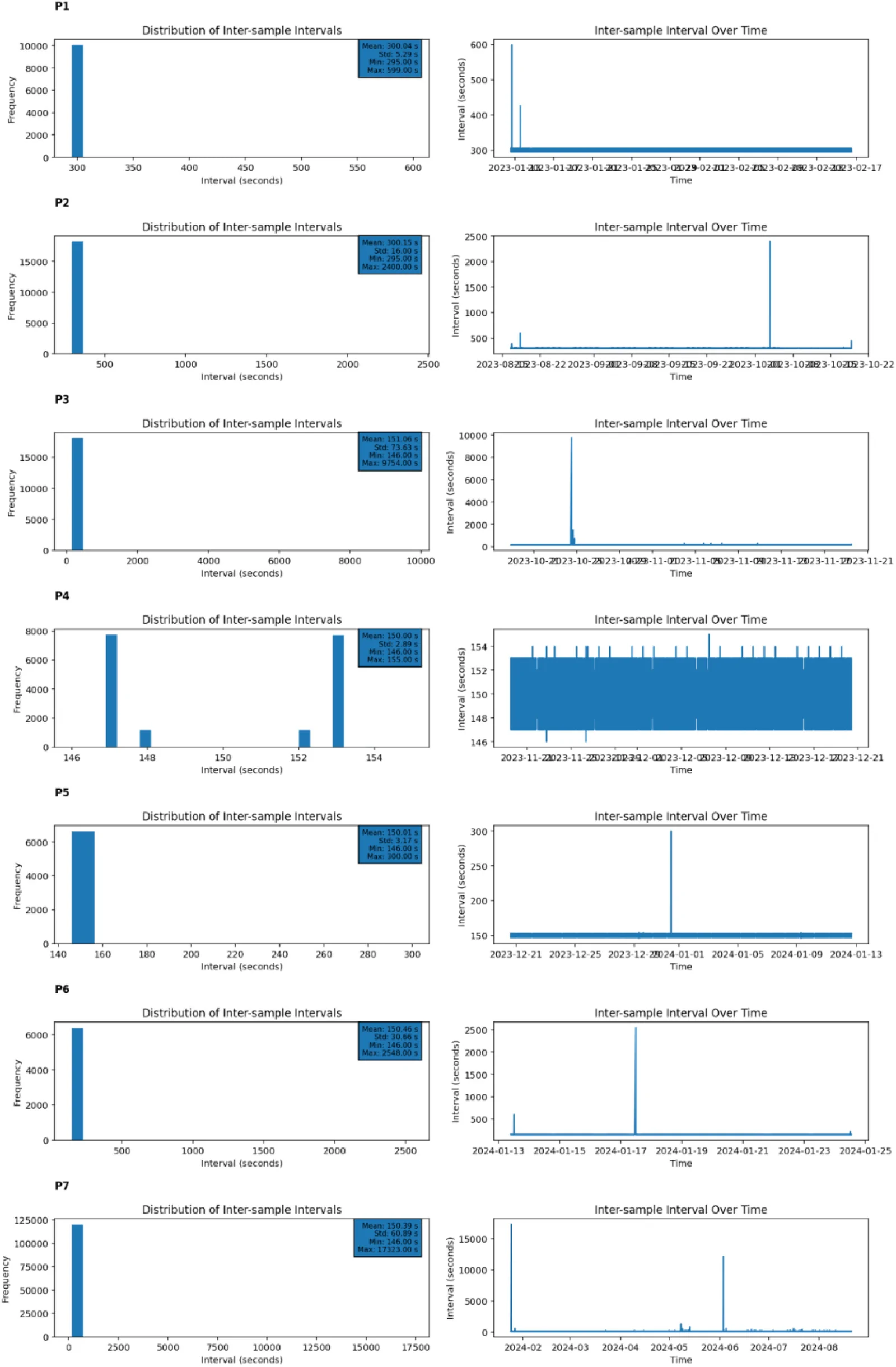
Inter-sample interval validation across all data collection periods.

**Local Variability and Noise Assessment:** Fig. 5 presents the rolling standard deviation of each environmental variable of P1 over time. Similar figures for the collection periods P2 to P7 are included in the data repository in *figures* → *technical_validation* folder. In Fig. 5, brighter colors indicate higher short-term variability and darker colors indicate more stable behavior. Clear differences can be observed across sensors. CO_2,_ light, and VOC show frequent bright vertical streaks, indicating repeated periods of rapid fluctuation. These spikes suggest dynamic environmental changes, likely driven by occupancy patterns, day and night-time cycles, or intermittent emission sources. In contrast, variables such as temperature, pressure, humidity, PM1, PM2.5, and PM10 remain mostly dark, reflecting relatively stable behavior throughout the monitoring period with only minor short-term fluctuations.

**Fig. 5:**
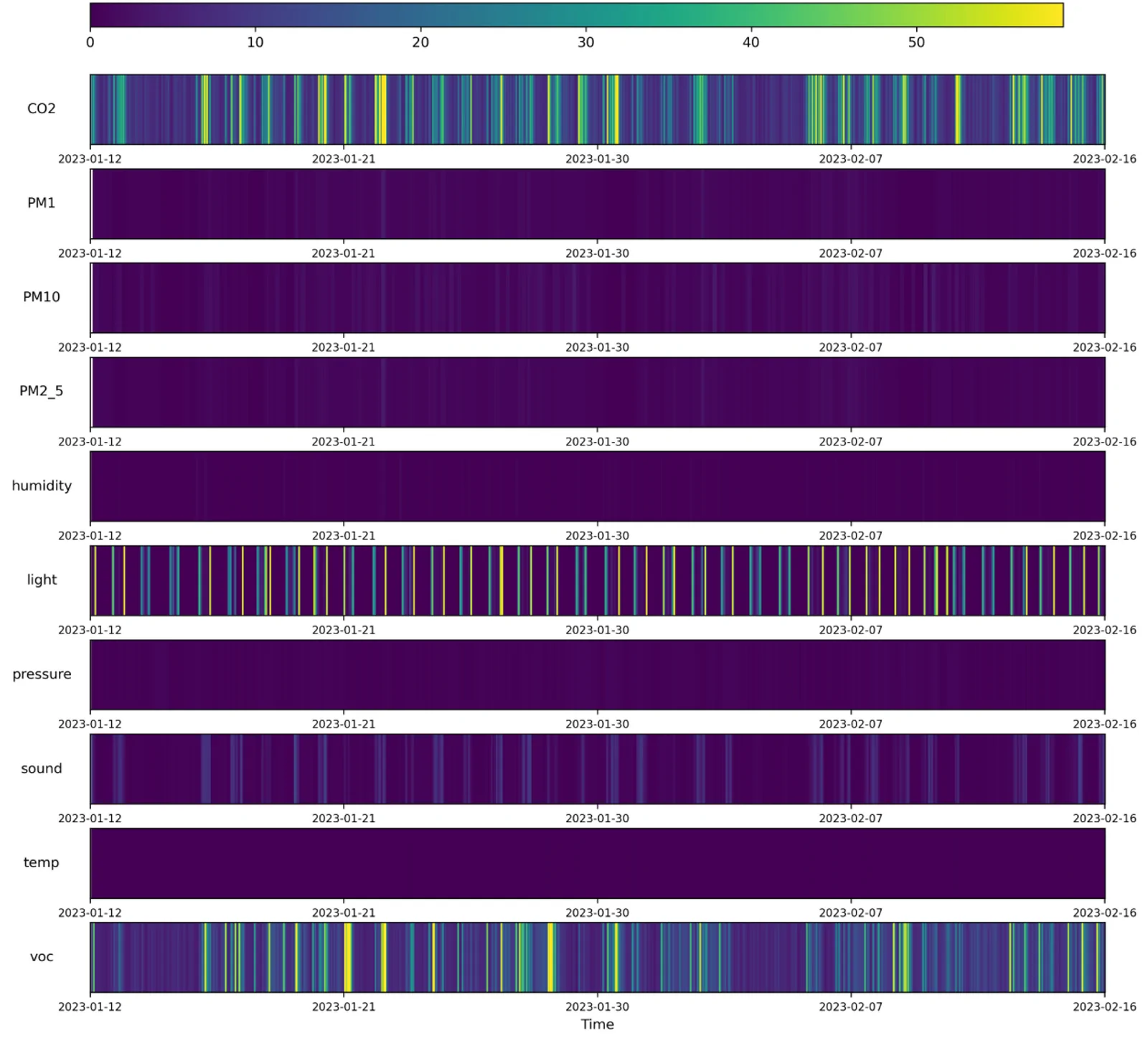
Heatmap of rolling standard deviation of the physical sensors of P1.

The temporal distribution of variability also provides insight into system behavior. The bright streaks in CO_2_, light, and VOC are spread across the entire time range rather than concentrated in a single interval, suggesting recurring events rather than isolated anomalies. Meanwhile, the near-uniform appearance of temperature and pressure indicates steady environmental control conditions. Overall, the heatmap highlights which variables are dynamically responsive and which remain stable, allowing quick identification of sensors that exhibit higher short-term variability and may warrant closer analysis in anomaly detection or environmental assessment studies.

**Cross Variable Consistency Check:** The correlation heatmap of the physical sensors for the first data collection period, i.e., P1, is shown in Fig. 6, whereas the correlation heatmaps for the rest of the data collection periods are made available in the data repository in *figures* → *technical_validation* folder. The correlation heatmap provides an overview of how the data variables relate to one another and serves as an important cross-variable consistency check within the technical validation process. The analysis was used to assess whether the observed relationships among sensors were consistent with expected environmental behavior.

**Fig. 6:**
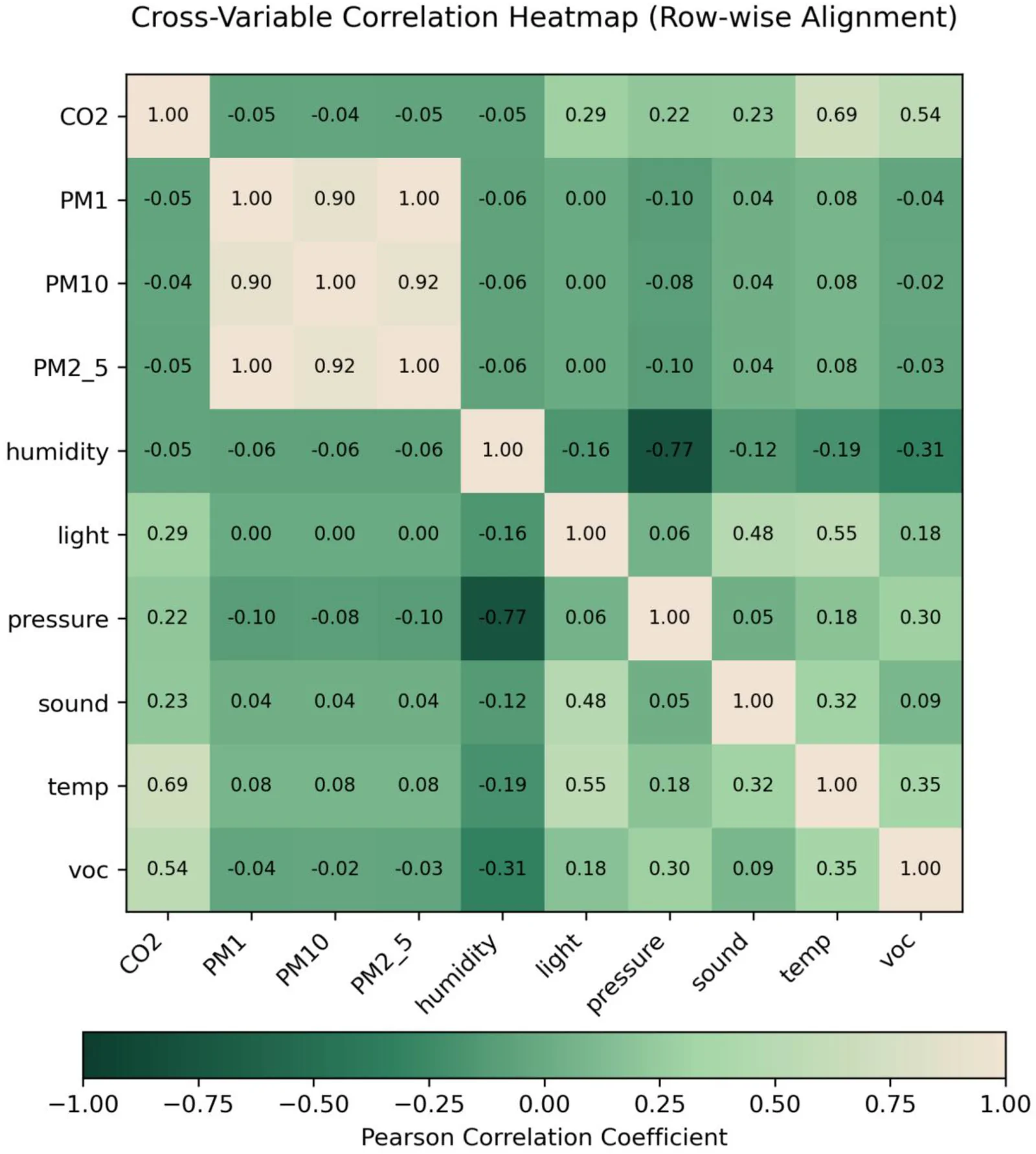
Cross-correlation heatmap of the physical sensors of P1.

Several strong and interpretable relationships are visible in the matrix. The particulate matter variables (PM1, PM2.5, and PM10) show very high positive correlations (i.e., ≈ 0.90–1.00), which is expected because these measurements represent overlapping particle size ranges and are typically influenced by the same emission sources. Temperature shows a strong positive correlation with CO_2_ (i.e., 0.69) and a moderate correlation with light (i.e., 0.55), which may reflect occupancy-driven patterns where human presence increases CO_2_ levels and lighting usage while also affecting indoor temperature. VOC also exhibits a moderate positive correlation with CO_2_ (i.e., 0.54), suggesting shared indoor sources such as human activity. A strong negative correlation between humidity and pressure (i.e., -0.77) is physically plausible, as atmospheric pressure variations often influence relative humidity levels. Meanwhile, weak correlations between particulate matter and most other variables suggest that particulate concentrations may vary somewhat independently of thermal or lighting conditions in this environment. Overall, the observed correlation patterns are consistent with known environmental behavior, supporting the internal consistency and integrity of the dataset and indicating that the sensors capture realistic and coherent environmental dynamics.

**Anomaly Severity Assessment:** To quantify anomaly severity, sensor observations within each labeled anomaly interval were compared with a period-level normal baseline constructed from observations outside the labeled anomaly intervals. Table 6 reports the baseline mean (μ), standard deviation (σ), mean absolute z-score (Mean |z|), maximum absolute z-score (Max |z|), percentages of observations exceeding 2 times σ (>2σ), and 3 times σ (>3σ). Higher absolute z-scores and larger exceedance percentages indicate greater and more sustained deviation from normal sensor behaviour. The results show that anomaly magnitude varied substantially across events and sensors. In P3, A2 showed a strong deviation in S2 (i.e., humidity), with a mean absolute z-score of 3.0 and 54.1% of observations exceeding 3σ. A3 produced sustained deviations in S3 (CO2), S8 (pressure), and S9 (sound), including 55.8% of S3 observations exceeding 2σ and all S8 observations exceeding 2σ. The largest deviations occurred during A7 in P6, where S1 (temperature), S2 (humidity), and S10 (light) exhibited mean absolute z-scores above 6σ, and all observations exceeded 3σ. Similarly, A8 in P6 and A11 in P7 showed noticeable S7 (VOC) and S5 (PM2.5) deviations, respectively. Only sensor–anomaly pairs showing substantial deviations are included in Table 6. Events with less or no substantial univariate deviation under the selected baseline, such as A4, A6, and A10, are retained in the data repository in the ‘*anomaly_severity_assessment.csv’ file* for transparency. Their lower univariate severity does not necessarily indicate that they are not anomalies, as some events may be contextual or arise from relationships among multiple variables rather than a large deviation in a single sensor.

**Table 6:** Anomaly severity assessment in different periods.

Period ID	Anomaly ID	Sensor ID	Baseline μ	Baseline σ	Mean |z|	Max |z|	>2σ (%)	>3σ (%)
P3	A1	S1	23.9	2.9	1.8	2.3	36.1	0
P3	A1	S2	54.2	9.2	1.6	2.2	38.9	0
P3	A1	S3	1436.6	451.3	1.7	2.1	38.9	0
P3	A2	S2	51.2	9.2	3.0	4.8	75.0	54.1
P3	A2	S5	82.3	110.4	1.7	5.7	29.2	8.3
P3	A3	S3	1436.6	451.3	2.4	5.7	55.8	40.8
P3	A3	S8	987.9	10.0	2.3	2.3	100.0	0
P3	A3	S9	51.2	11.9	1.9	2.8	61.7	0
P4	A5	S2	69.5	8.5	2.9	3.7	68.3	60.0
P4	A5	S3	1461.4	487.1	1.9	2.1	60.0	0
P6	A7	S1	1.8	8.5	6.6	7.7	100.0	100.0
P6	A7	S2	68.6	6.0	6.6	7.3	100.0	100.0
P6	A7	S3	1609.1	452.4	2.2	2.3	95.8	0
P6	A7	S10	1.9	8.6	6.5	7.6	100.0	100.0
P6	A8	S7	287.8	330.1	3.7	5.2	79.2	70.8
P6	A9	S2	68.6	6.0	2.4	3.3	59.2	53.1
P6	A9	S3	1609.1	452.4	2.4	2.7	97.9	0
P7	A11	S2	44.0	8.8	2.0	4.3	62.5	4.2
P7	A11	S5	42.8	103.1	2.8	7.1	45.8	41.7
P7	A11	S7	194.3	198.3	3.4	8.5	50.0	41.7

**Statistical Screening of Candidate Naturally Occurring Deviations:** In addition to the predefined contextual anomaly intervals, we conducted an exploratory statistical screening to identify sensor observations that deviated markedly from their local temporal behaviour. This procedure was intended to identify *candidate* naturally occurring deviations for documentation and further interpretation; it does not establish that every flagged interval represents a true anomaly, fault, or unusual real-world event. For each sensor time series, a local robust baseline was estimated from neighbouring non-contextual observations using the median and median absolute deviation (MAD). Each observation was then expressed as a robust z-score relative to this local baseline. Observations with an absolute robust z-score of at least 3.5 were recorded as statistical candidates. An isolated deviation was labelled a point candidate, whereas consecutive deviations satisfying the predefined persistence criterion were labelled a collective candidate. Intervals already documented as contextual anomalies were excluded before screening.

The resulting CSV of this statistical screening is included in the main data repository in ‘*candidate_unknown_anomalies.csv’* file. This CSV file reports the period, sensor, start and end timestamps, anomaly type, number of observations, direction of deviation, robust-z-score summaries, median, and median absolute deviation. This file should be interpreted as a transparent record of statistically unusual sensor behaviour rather than as definitive anomaly labels. In particular, several candidates may reflect routine household activities, such as cooking, occupancy-related changes, or normal environmental variation, rather than anomalous events. Accordingly, we use the term “candidate naturally occurring deviation” throughout, because this classification is based solely on the magnitude and persistence of deviation from the local robust baseline.

## 4 Experimental Design, Materials and Methods

This section describes the data collection setup, collection environments, deployment details, and procedures used to generate the contextual anomaly data.

### 4.1 Data collection setup

The data collection was performed using smart devices manufactured by Airthings [22]. As described in Table 7, two devices are used for data collection and transmission, i.e., Airthings View Plus and Airthings Space Hub. View Plus, as shown in Fig. 7, comprises multiple sensors within the same device. View Plus can run on batteries or can be plugged into a power adapter using a Universal Serial Bus Type-C (USB-C) cable. When View Plus runs on batteries, the default data interval is 5 minutes, whereas when it runs on USB-C, the interval can be chosen to be either 2.5 or 5 minutes. The other main component of the data collection setup, i.e., Airthings Space Hub, is illustrated in Fig. 8. The power source of the Space Hub is an adapter, and it connects to the internet via an Ethernet cable.

**Table 7:** Devices details.

No.	Device Name	Connectivity	Power
1	View Plus	Airthings SmartLink	Batteries / USB-C
2	Space Hub	Ethernet	Adapter

**Fig. 7:**
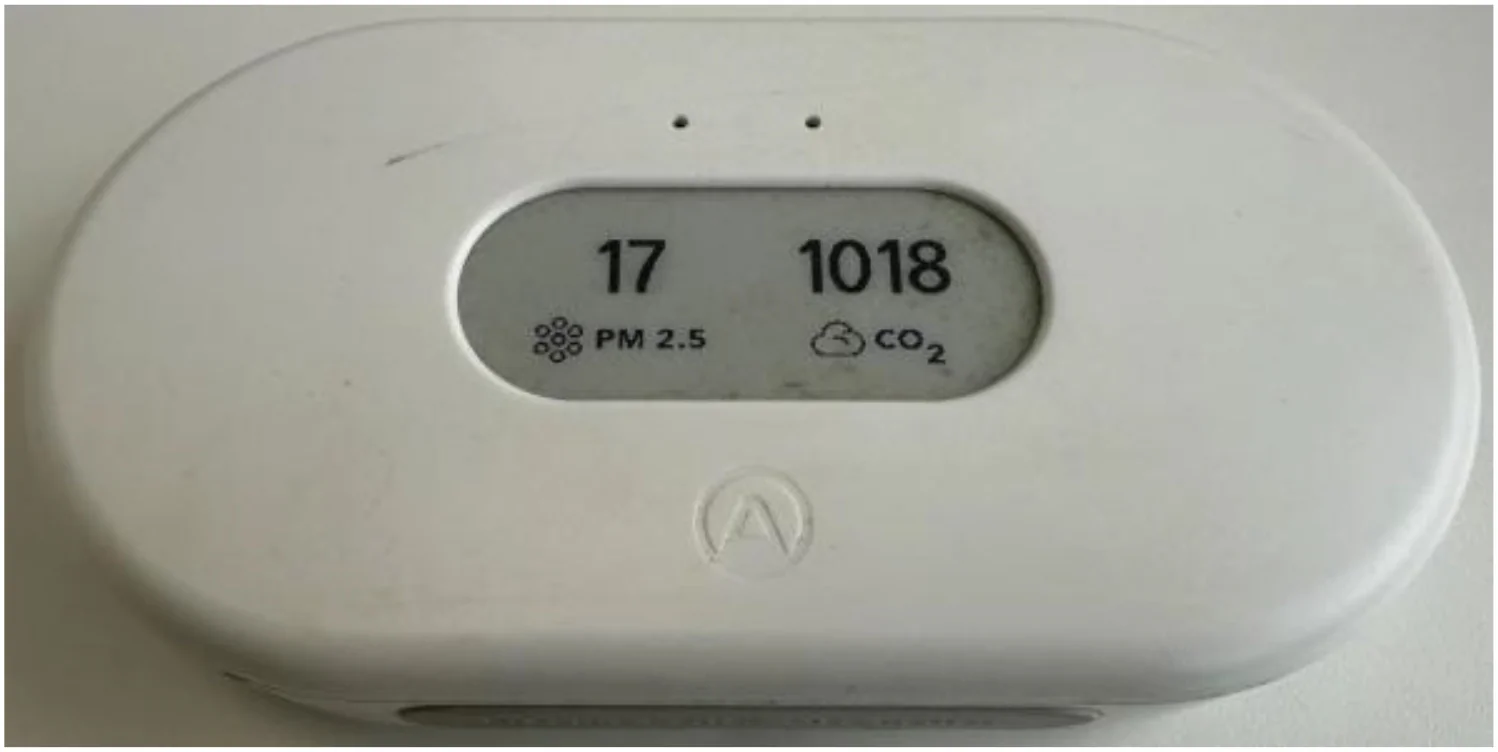
Airthings View Plus device.

**Fig. 8:**
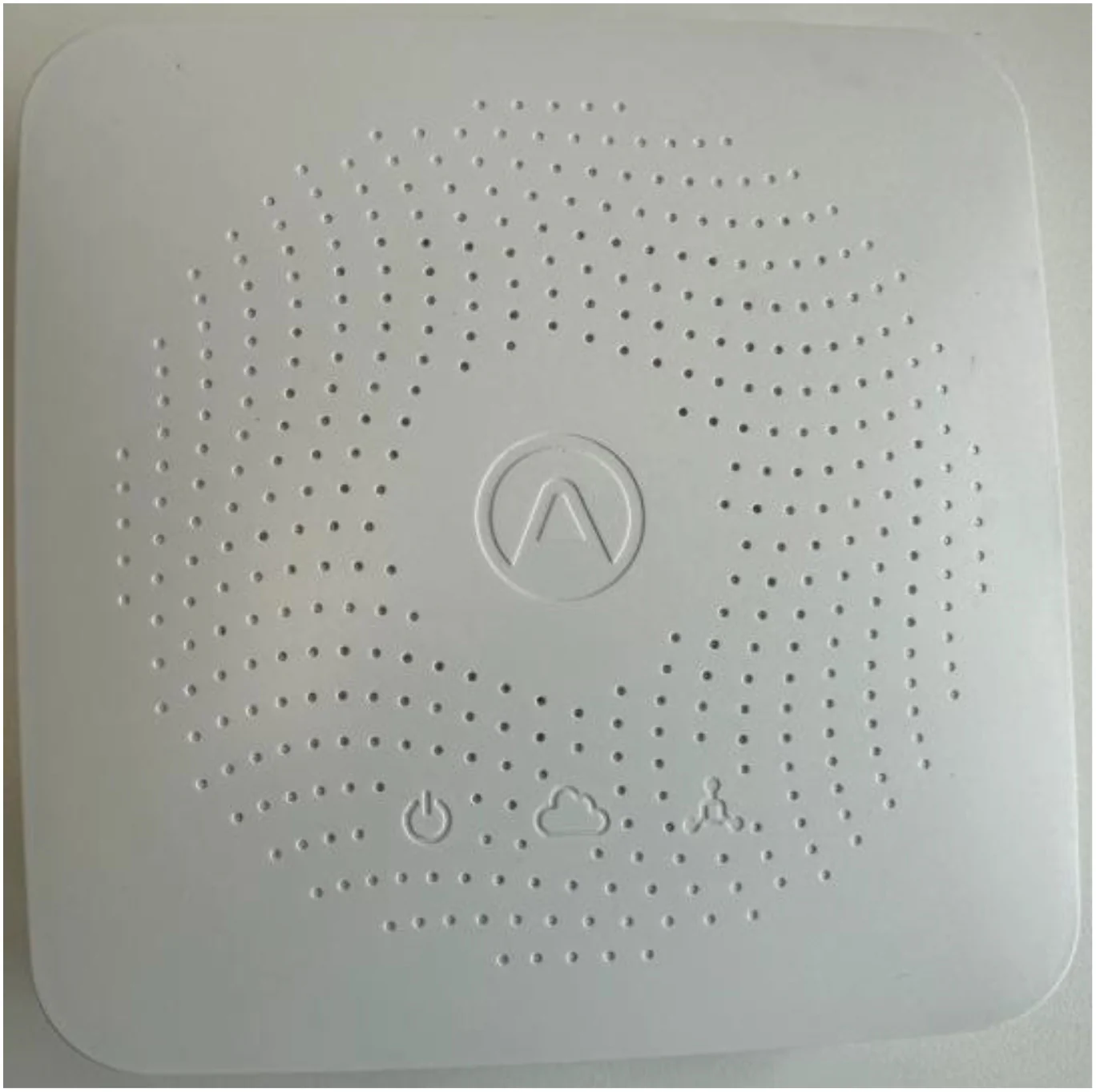
Airthings Space Hub device.

Fig. 9 depicts the components in the data collection setup. View Plus collects data using sensors that measure air quality, light, and sound, and transmits the data to the Space Hub via the Airthings SmartLink protocol. The Space Hub then sends data to the cloud server over the internet, which is subsequently visualized on the dashboard. The data can be downloaded from the dashboard or by consuming the Application Programming Interface (API), in the form of Comma-Separated Values (CSV) files for a specified period.

**Fig. 9:**
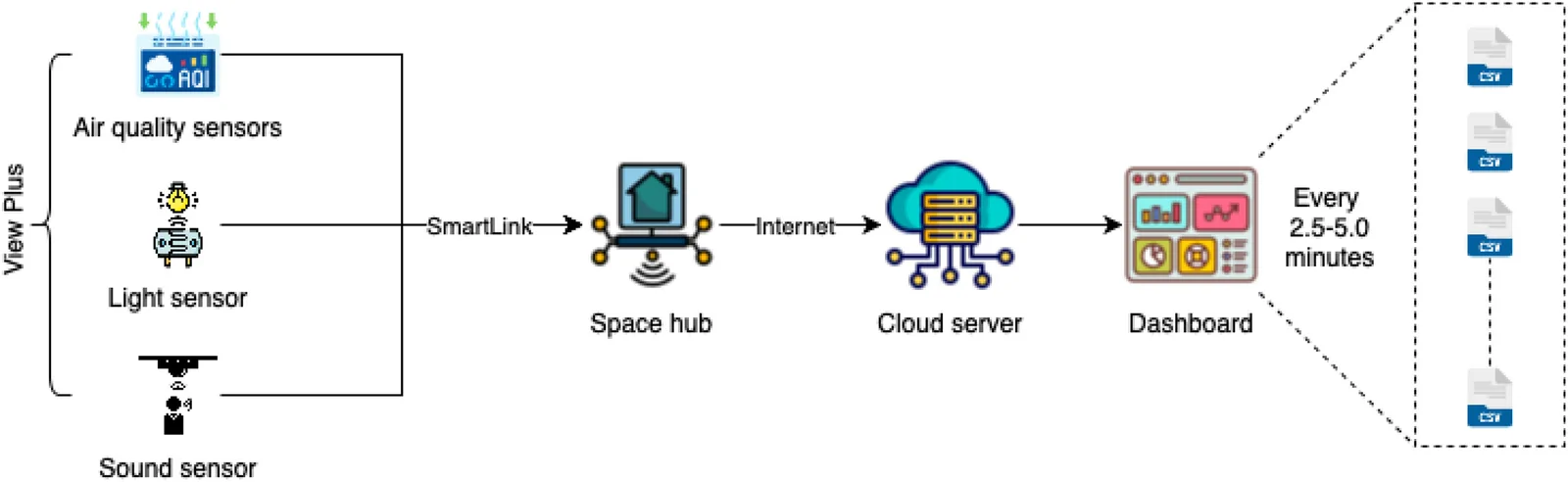
Components of the data collection setup.

### 4.2 Data collection environment

The datasets in SHoRT-CA were collected in Gjøvik, Norway, at three different locations. Table 8 provides details of the different locations where data were collected. The first location, referred to as L1-Office, is situated on the first floor of a building on the premises of the Norwegian University of Science and Technology. Fig. 10 shows the floor plan of the office. It has one window, one door, and a Heating, Ventilation, and Air Conditioning (HVAC) system is in place to manage airflow and air quality. The View Plus device was placed 1.3 meters above the floor, lying flat on a table, and facing the ceiling. During the time of data collection, 2-3 adults were present in the office. The other two locations, i.e., L2-Kitchen and L3-Bedroom, are part of a Norwegian household located on the second floor of a house that was built in the 1970s. Fig. 11 shows the floor plan of the second floor of the house. There were two adults and two children living in the house during the data collection. For L2-Kitchen, the View Plus device was placed in the kitchen, specifically, on top of the hood above the stove, 2 meters above the floor and facing the ceiling. The kitchen features natural ventilation through doors and windows, as well as an exhaust fan, which is utilized during cooking activities. L3-Bedroom is in the same house, but only has natural ventilation in the form of a window and a door. The View Plus device in L3-Bedroom was placed on the floor in the corner of the room, facing the ceiling. During the normal collection periods, the environments were used under their ordinary conditions rather than under a fixed experimental schedule. Routine activities such as cooking, occupancy, ventilation, and day/night transitions were allowed to occur naturally outside the planned anomaly experiments.

**Table 8:** Details of the three locations.

Location ID	Floor	No. of Windows	No. of Doors	Ventilation
L1-Office	1	1	1	HVAC
L2-Kitchen	2	3	2	Natural and exhaust
L3-Bedroom	2	1	1	Natural

**Fig. 10:**
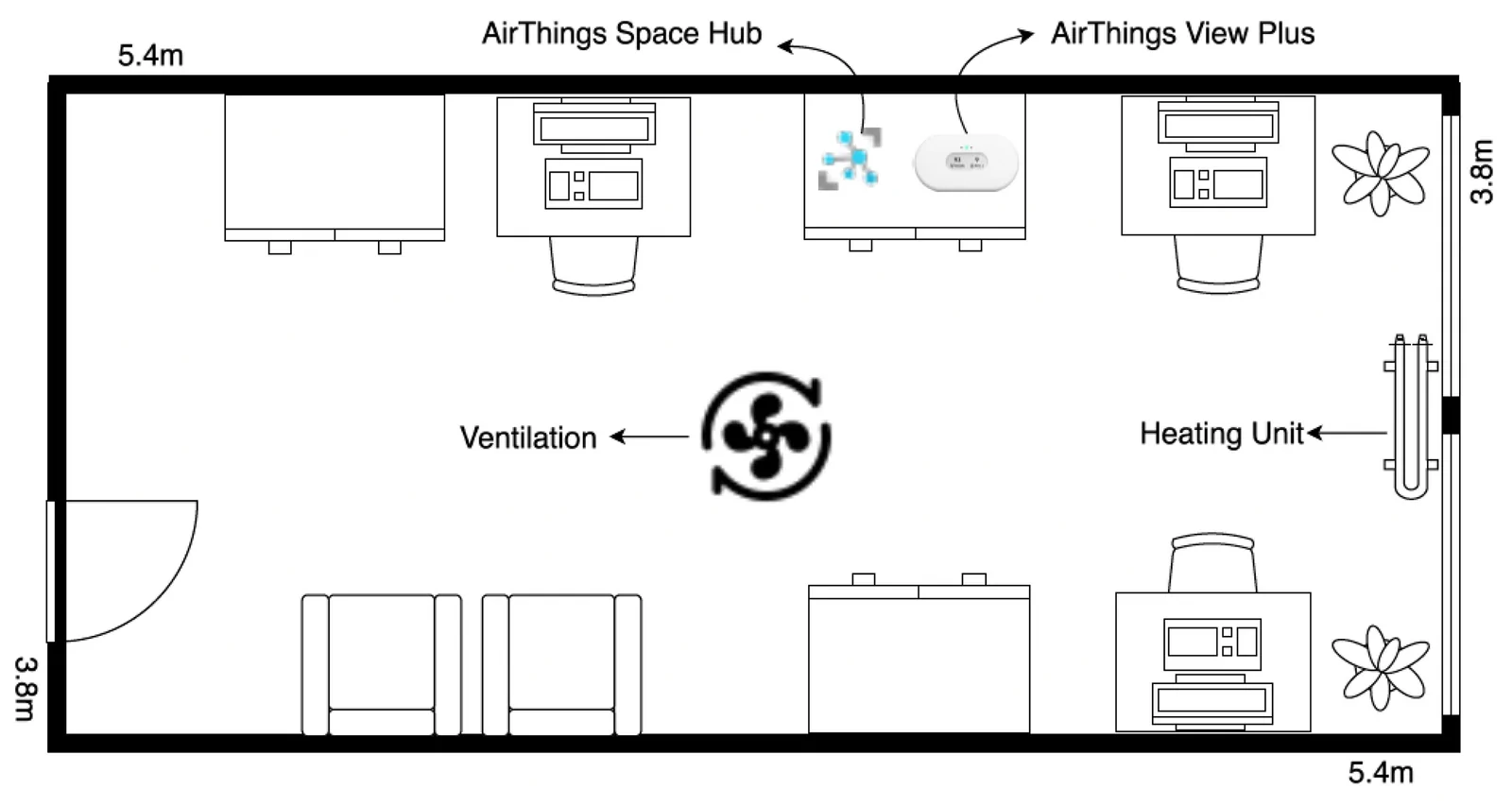
Floor plan of L1-Office.

**Fig. 11:**
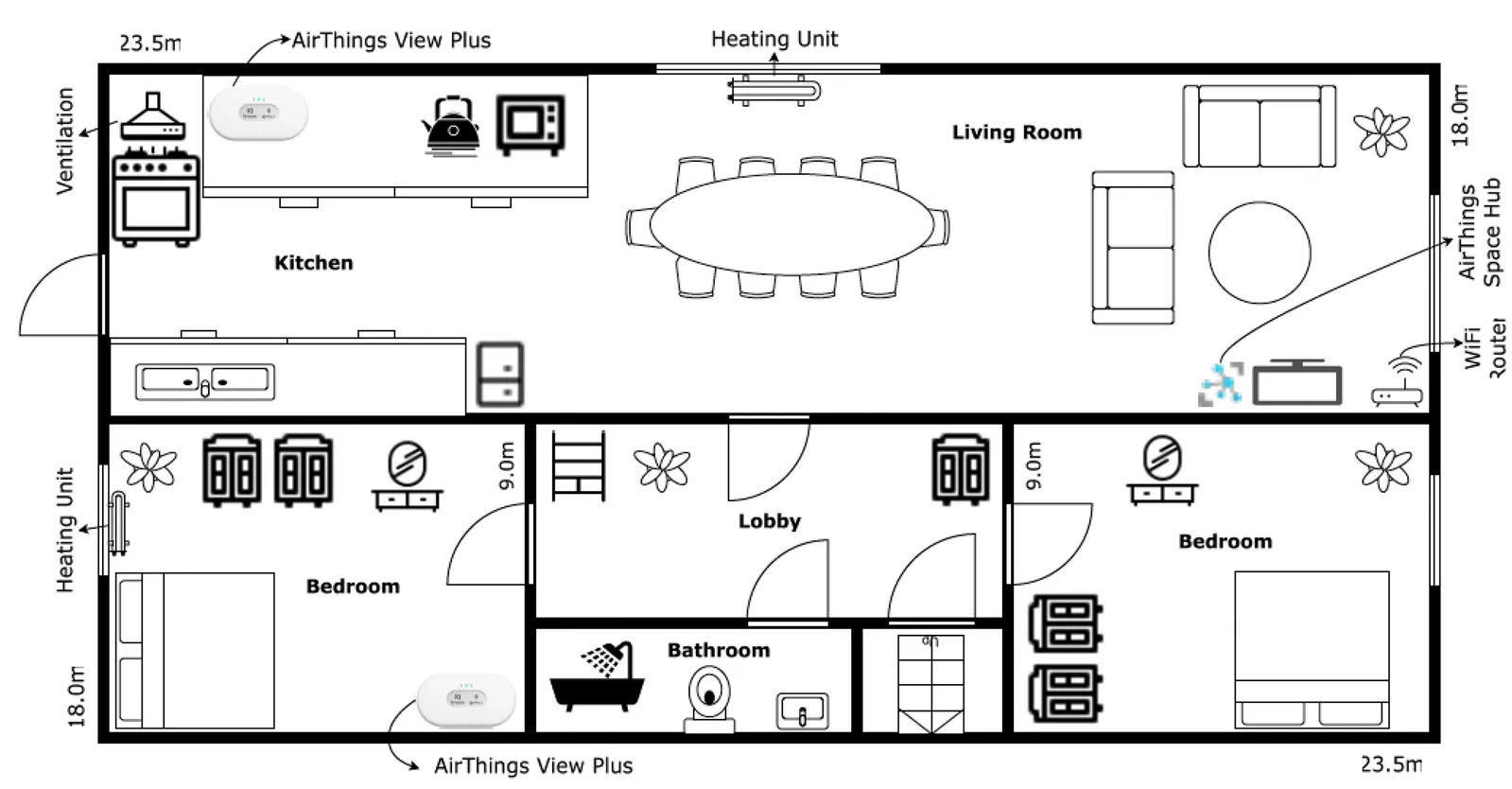
Floor plan of L2-Kitchen and L3-Bedroom.

### 4.3 Deployments

Table 9 provides the details of deployed sensors and their respective measuring units and calibration periods. There are two types of sensors, i.e., physical and virtual. Ten physical sensors are installed inside the View Plus device, and they record temperature, humidity, CO_2_, PM1, PM2.5, PM10, VOC, pressure, sound, and light from the surroundings. The temperature, humidity, PM1, PM2.5, PM10, pressure, sound, and light sensors are automatically calibrated, and they give accurate readings right after they are set up. The CO_2_ and VOC sensors require a 7-day calibration period, after which their readings become accurate [23]. The virtual sensors are calculated values based on physical sensors, and they measure virus risk, occupants, and presence. The virtual sensors were included to provide contextual variables that complement the physical environmental measurements. Occupancy and presence provide information about human activity and temporal context, which can help distinguish normal environmental variations associated with occupancy from potentially anomalous conditions. The virus risk sensor provides a derived measure combining environmental and occupancy-related factors. Occupancy and presence are computed by analyzing the room temperature and CO_2_ levels over time. Virus risk is computed using the readings of temperature, CO_2_, and humidity, considering four factors, i.e., virus survival rate, room occupancy, ventilation rate, and transmission efficiency. Since virtual sensors require values from the CO_2_ sensor, the calibration time of virtual sensors is the same as that of the CO_2_ sensor, i.e., 7 days.

**Table 9:** Details of the physical and virtual sensors.

Sensor ID	Sensor Name	Type	Units	Calibration Period
S1	Temperature	Physical	°C	Automatic
S2	Humidity	Physical	%	Automatic
S3	Carbon Dioxide (CO_2_)	Physical	ppm	7 days
S4	Particulate Matter (PM) 1	Physical	μg/m^3^	Automatic
S5	Particulate Matter (PM) 2.5	Physical	μg/m^3^	Automatic
S6	Particulate Matter (PM) 10	Physical	μg/m^3^	Automatic
S7	Volatile Organic Compound (VOC)	Physical	ppb	7 days
S8	Pressure	Physical	hPa	Automatic
S9	Sound	Physical	dB SPL	Automatic
S10	Light	Physical	%	Automatic
S11	Virus Risk	Virtual	0-10	7 days
S12	Occupants	Virtual	count	7 days
S13	Presence	Virtual	minutes	7 days

As shown in Table 9, each sensor is given an ID, e.g., S1, S2, S3, etc. Sensors will be referred to by their respective IDs in the rest of this paper. The datasets comprise multiple data collection periods depending on the timeline and location. Table 10 describes the period ID, start and end timestamps, season, and location ID. There are a total of seven collection periods, i.e., P1 to P7, between 2023-01-12 and 2024-08-20. Three collection periods, i.e., P1, P2, and P5, are recorded at the office, two collection periods, i.e., P3 and P7, are recorded at the kitchen, and two collection periods, i.e., P4 and P6, are recorded at the bedroom. Table 10 also mentions the seasons during the collection periods. This is important because outside weather and air quality greatly affect the indoor air quality. For example, during the winter, outside temperatures in Gjøvik can drop to -30°C, which results in higher use of heating with minimal ventilation. Similarly, during spring/summer, there is no heating inside, but there is a higher pollen count outside, which affects the indoor air quality when the windows are opened.

**Table 10:** Details of the data collection periods.

Period ID	Start Date	End Date	Season	Location ID
P1	2023-01-12	2023-02-16	Winter	L1-Office
P2	2023-08-16	2023-10-18	Summer/Autumn	L1-Office
P3	2023-10-18	2023-11-19	Autumn	L2-Kitchen
P4	2023-11-19	2023-12-20	Autumn/Winter	L3-Bedroom
P5	2023-12-20	2024-01-12	Winter	L1-Office
P6	2024-01-13	2024-01-24	Winter	L3-Bedroom
P7	2024-01-24	2024-08-20	Winter, Spring, Summer	L2-Kitchen

## Limitations

The data were collected from three indoor environments, i.e., an office, a kitchen, and a bedroom, within the same geographical region, which represent common indoor settings but do not capture the full diversity of building types, climates, or smart home configurations. In addition, the dataset originates from a single smart home and office deployment, and the contextual anomalies were generated through semi-controlled experiments designed to mimic realistic events. As a result, while the dataset provides realistic sensing scenarios for methodological research, results obtained from it may require further validation in larger and more diverse real-world deployments. All sensors do not share the same data interval because sensors measuring VOC and pressure have a 5-minute interval, and a virtual sensor measuring virus risk has a 60-minute interval by design, whereas all other sensors have either a 2.5- or 5-minute interval, depending on their energy source. Hence, there is a difference in the total number of instances of each sensor for each available data period. Also, not all of the sensors’ data is available for all data periods. Future extensions of the repository may include additional deployment locations and longer monitoring periods to further increase environmental diversity and support broader generalization studies.

## Ethics Statement

This study was reviewed by the data protection and research ethics office of the Norwegian University of Science and Technology (NTNU), Gjøvik, Norway. It was determined that formal ethical approval from an institutional review board is not required for the collection of these data, as the dataset does not contain personally identifiable information and does not qualify as sensitive human data under applicable Norwegian regulations. All occupants and guests of the residential locations (L2-Kitchen and L3-Bedroom) were informed of the data collection and provided their consent for participation and data sharing. For the office space (L1-Office), the data collection was conducted within the premises of NTNU, and approval for data collection was granted by the department responsible for the space.
